# Nanostructure design for drastic reduction of thermal conductivity while preserving high electrical conductivity

**DOI:** 10.1080/14686996.2017.1413918

**Published:** 2018-01-12

**Authors:** Yoshiaki Nakamura

**Affiliations:** ^a^ Graduate School of Engineering Science, Osaka University, Toyonaka, Japan

**Keywords:** Nanodots, nanostructures, silicon, germanium, molecular beam epitaxy, thermal conductivity, thermoelectric materials, electron transport, phono transport, 10 Engineering and Structural materials, 101 Self-assembly / Self-organized materials, 201 Electronics / Semiconductor / TCOs, 206 Energy conversion / transport / storage / recovery, 210 Thermoelectronics / Thermal transport / insulators, 212 Surface and interfaces, 302 Crystallization / Heat treatment / Crystal growth, 306 Thin film / Coatings

## Abstract

The design and fabrication of nanostructured materials to control both thermal and electrical properties are demonstrated for high-performance thermoelectric conversion. We have focused on silicon (Si) because it is an environmentally friendly and ubiquitous element. High bulk thermal conductivity of Si limits its potential as a thermoelectric material. The thermal conductivity of Si has been reduced by introducing grains, or wires, yet a further reduction is required while retaining a high electrical conductivity. We have designed two different nanostructures for this purpose. One structure is connected Si nanodots (NDs) with the same crystal orientation. The phonons scattering at the interfaces of these NDs occurred and it depended on the ND size. As a result of phonon scattering, the thermal conductivity of this nanostructured material was below/close to the amorphous limit. The other structure is Si films containing epitaxially grown Ge NDs. The Si layer imparted high electrical conductivity, while the Ge NDs served as phonon scattering bodies reducing thermal conductivity drastically. This work gives a methodology for the independent control of electron and phonon transport using nanostructured materials. This can bring the realization of thermoelectric Si-based materials that are compatible with large scale integrated circuit processing technologies.

## Graphical abstract

**Figure:**
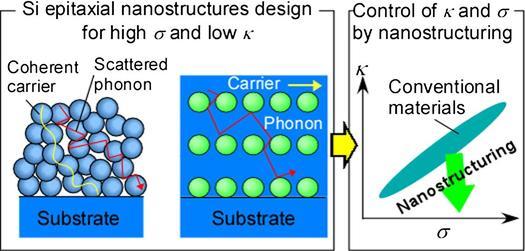

## 1. Introduction

The nanostructure science has received significant attention because nanostructured materials can exhibit novel characteristics that the bulk materials do not show. Of particular note are the properties related to quantum confinement effects. These effects have gone beyond academic interest with nanostructured materials now being used in a range of industrial fields. The science of this field has contributed to the progress of electronic and optical devices and their systems [1–3], which has had significant impacts on high level information society.

Although electronic and optical technologies have developed significantly, the control of the thermal properties of materials is less advanced. However, the importance of thermal management is recognized, and thus novel materials, devices and systems related to thermal management are required to be developed. Therefore, nanostructured materials are expected to progress this field, as they have for electronic and optical devices. Control of charge carriers and photon transport is important in electronic and optical devices, while the key for thermal flow control is often phonon transport although electronic carriers can also affect heat transport. Phonon transport in materials that contain structures, such as grains, is often treated as quasi-particle transport because of the small coherent length. Recently, nanostructured materials have exhibited thermal conductivities that could not be explained using conventional theory [4–7]. The importance of research on phonon scattering and transport in nanostructured materials is recognized, with recent academic interest in examining the wave properties of phonons [6–8]. In addition to the scientific significance of understanding phonon transport in nanostructured materials, this knowledge will improve thermal management technologies.

**Figure 1.**
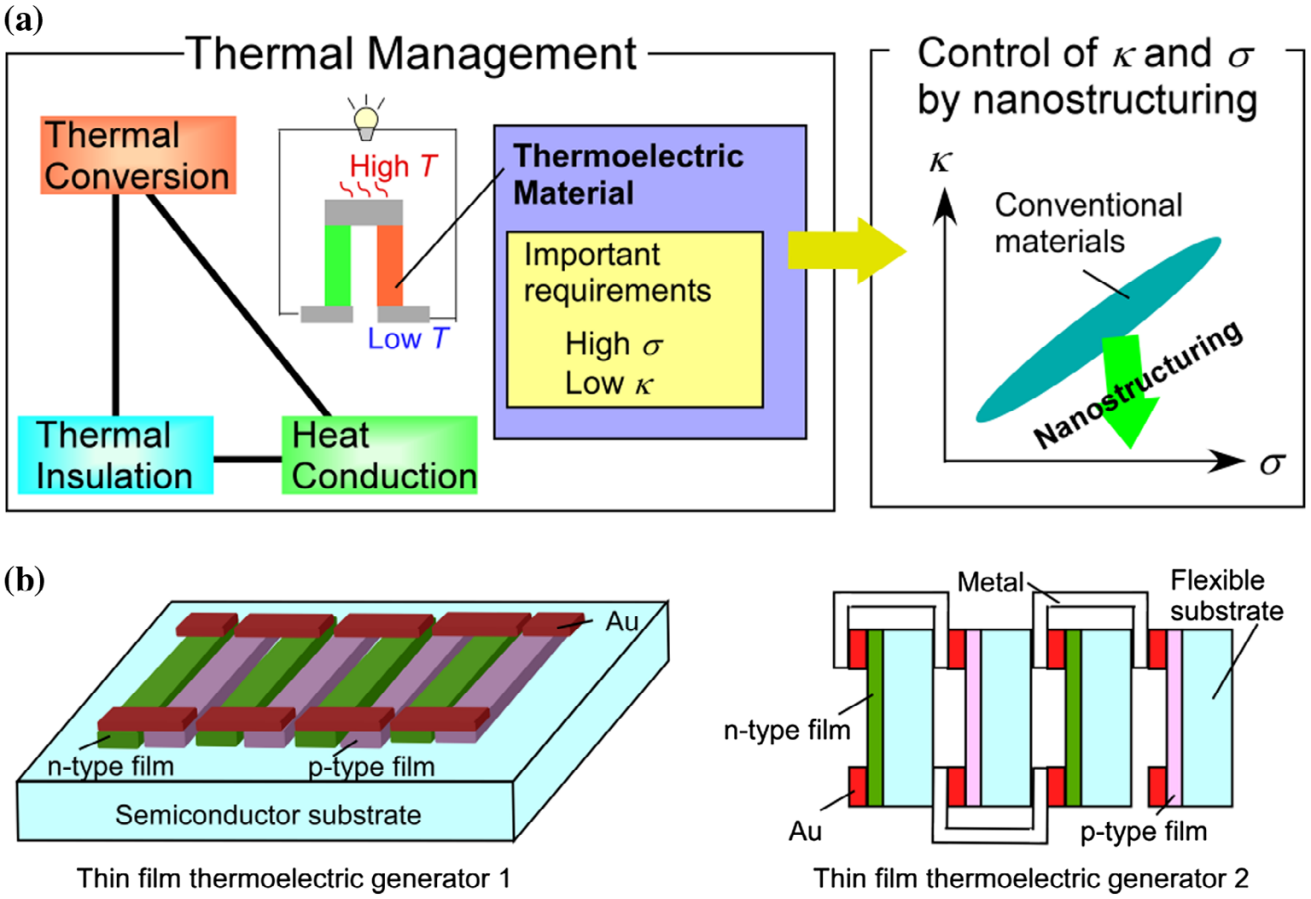
(a) Importance of independent control of thermal and electric conductivities by nanostructuring in thermoelectric material study. (b) Examples of thin film thermoelectric generators.

Thermoelectric conversion is an important item for thermal management (Figure 1) and offers the possibility for waste heat to be reused as electrical energy. This can be an ideal energy source. The issue in this thermoelectric conversion, which is a function of temperature difference and the dimensionless figure of merit (*ZT*) determined by a material, is an insufficient efficiency. *ZT* is described as *S* ^2^ *σT*/*κ*, where *S* is Seebeck coefficient, *σ* is electrical conductivity, *κ* is thermal conductivity, and *T* is the absolute temperature. To enhance the thermoelectric conversion efficiency of a material, the value of *ZT* must be increased and thus materials with low thermal conductivity and high electrical conductivity are required. Conventionally, to realize this, heavy elements were used for low thermal conductivity [9,10]. In general, however, heavy elements are often rare elements, the use of which prevents the industrial application to thermoelectric materials. Recently, introduction of nanostructures that can reduce the thermal conductivity is expected as a promising approach for the realization of rare-element-free thermoelectric material. Therefore, the thermal physics of phonon transport in nanostructured materials must be understood to allow for independent control of electrical and thermal transport, which will lead to non-conventional thermoelectric materials with high efficiency.

In this review, we outline a methodology for the independent control of phonon and electron transport in the materials that have a large phonon contribution to heat transport. This is achieved using nanostructure formation technologies (Figure 1). Strategies for the independent control of phonon and electron transport and the nanostructure formation technologies are introduced in Section 2. Examples are given in Sections 3 and 4, and the results are summarized in Section 5.

## 2. Strategy for thermal and electrical control using nanostructure design and formation technologies

### 2.1. The strategy and the application to thermoelectric materials

Phonons are generally involved in heat conduction in thermoelectric materials. The introduction of nanostructures in a material [11–21] causes the phonons to be scattered at their interfaces, which lowers the thermal conductivity. Therefore, the conventional strategy to lower the thermal conductivity of a material involves making smaller nanostructures to increase interface density, which leads to greater phonon scattering [22]. This conventional approach assumes that the interfacial thermal resistance related to the probability of phonon scattering does not depend on the curvature of the interface, but does depend on the material. Therefore, when these properties of a material are determined, the interfacial thermal resistance is assumed to be constant. However, we expect that in the case that the introduced structure size are miniaturized to be nanometer scale, the phonon scattering probability (or transmission probability) itself at one interface (not scattering frequency) can increase (or decrease) by changing the interface curvature radius. Namely, the interface curvature radius changes interfacial thermal resistances.

In this way, the thermal conductivity of a material is lowered by introducing nanostructures. Furthermore, if electric conductivity can be increased at the same time, this approach can be used to develop high-performance thermoelectric materials. As mentioned previously, the *ZT* for thermoelectric conversion is defined as *S* ^2^ *σT*/*κ*, with the power factor of thermoelectric conversion being described by *S* ^2^ *σ.* The introduction of nanostructures generally does not affect *S* so much except in two-dimensional structures such as two-dimensional electron or hole gasses [23]. Although the development of materials with high electrical and low thermoelectric conductivities is critical, these properties are generally correlated. Thus, the independent control of electric and thermal conductivities is a vital goal for long.

In this review paper, we describe a methodology to fabricate materials with high carrier conductivity and low thermal conductivity by incorporating nanostructures with well-controlled interfaces at the atomic level. We have focused on Si-based materials, which are both environmentally friendly and inexpensive. Typically, Si is not an effective thermoelectric material because it has a high thermal conductivity, despite its high power factor for thermoelectric conversion [24–26]. Therefore, if the thermoelectric conductivity of Si can be reduced while retaining a high power factor, the abundant waste heat generated from Si-based, large-scale integrated circuits (LSI) could be reused as electrical energy. Many thermoelectric generators using films [27–29] are proposed recently as shown in Figure 1(b). Such modules or devices can be applied to various digital devices related to LSI if Si-based thermoelectric materials are realized. It indicates that its realization can give a useful energy source for a lot of sensors connected to LSI in oncoming ‘internet of things’ society. Because Si processing technologies are mature, Si-based thermoelectric materials will be highly attractive to industry. Then, aforementioned industrial use of such waste heat could be achieved using Si nanomaterials with high thermoelectric performance.

**Figure 2.**
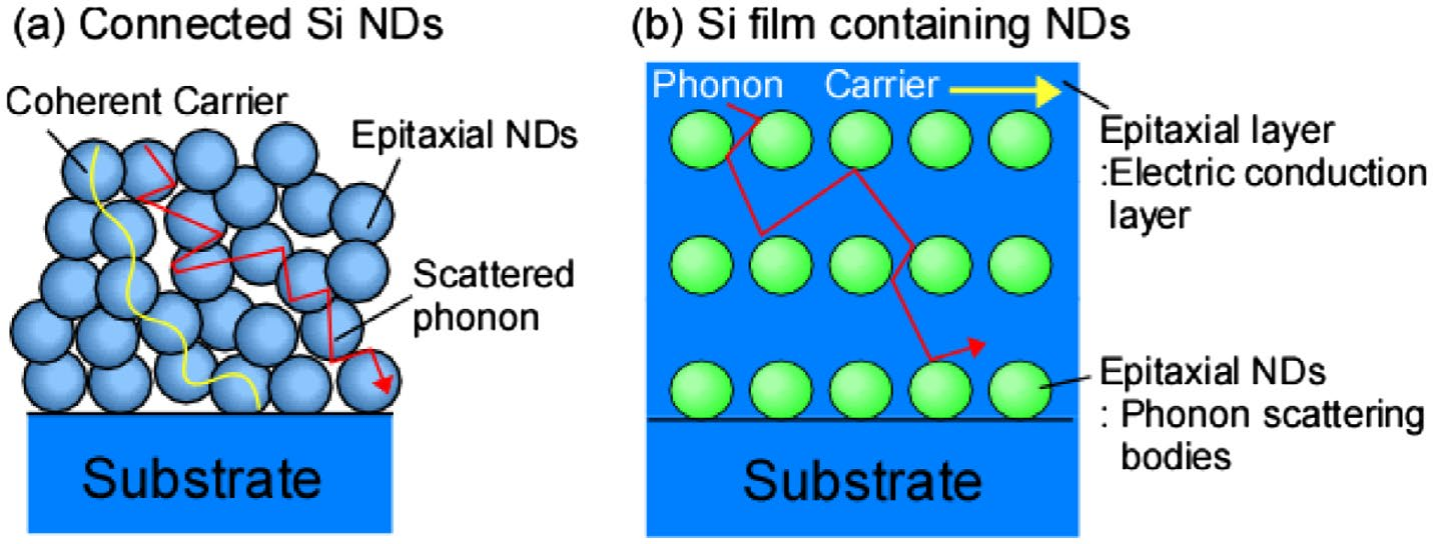
Proposed nanostructures for independent control of thermal and electric conductivities. (a) structure of connected Si NDs and (b) Si film containing NDs.

Here, we propose the two Si-based nanostructures for the independent control of electric and thermal conductivities (retaining high electric conductivity and reducing thermal conductivities), as shown in Figure 2 [30,31]. One structure is connected nanometer-sized epitaxial Si NDs that all had the same crystal orientation. We expect that the wavefunction of the charge carriers in this material should spread coherently because of the ordered crystal orientation of the NDs. Conversely, phonons with a long mean free path are easily scattered at the ND interfaces. Thus, the high electrical conductivity should be retained while the thermal conductivity should decrease [30]. The alternative structure consisted of Si films containing nanometer-sized epitaxial Ge NDs. The Si layer and the Ge NDs should serve as the electrical conduction layer and phonon scattering bodies, respectively. Because the separate structures have separate roles, we expected independent control of the electrical and thermal conductivities [31]. The density of the ND interfaces and their size were correlated within the structure of the connected Si NDs. However, the two components were controlled independently in the structure of the Si films containing Ge NDs, which allowed greater controllability.

As discussed previously, there is great value, both academically and practically, in the fabrication of arbitrary nanostructures and bringing out novel material properties on the basis of nanostructural physics. Because the physics of heat transport within nanostructures is not yet fully understood, the structures that form the interfaces and influence materials properties must be well-defined (shape, crystal structure, composition, strain etc.). However, controlling the purity, crystal structure uniformity, interface shape, and composition of a nanomaterial is difficult when the fabrication method for bulk materials is used. These technical difficulties hinder the understanding of the material physics at the nanoscale. Thus, the fabrication using highly controlled nanotechnologies is required to clarify the theory of phonon transportation on the nanoscale, which will in turn result in progress in the field.

### 2.2. Nanostructure formation technology

**Figure 3.**
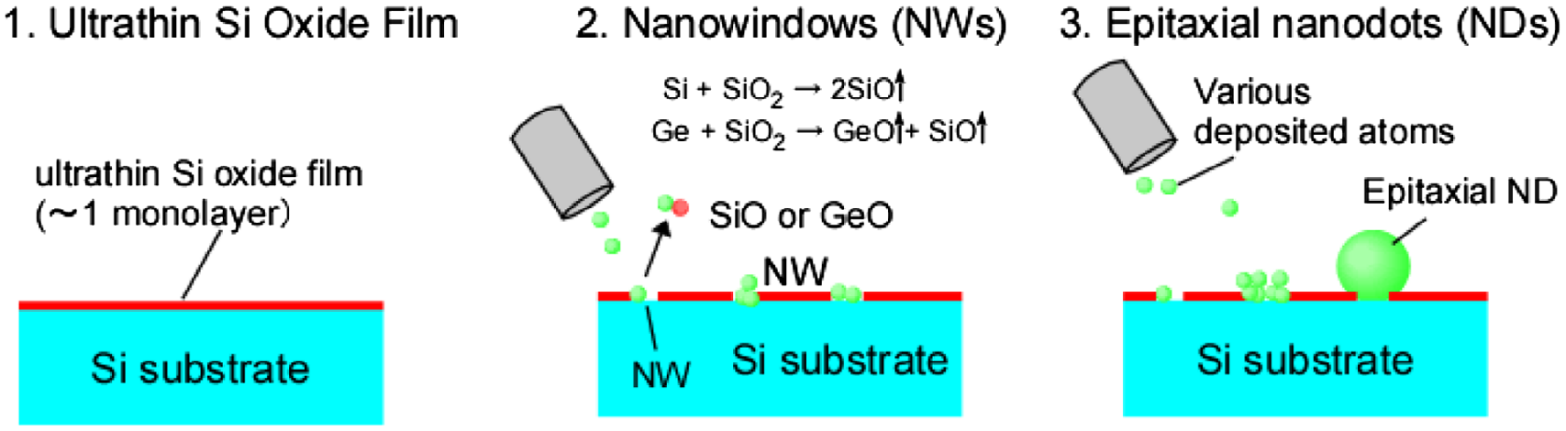
Ultrathin Si oxide film technique for epitaxial growth of NDs.

‘Ultrathin’ Si oxide films with a thickness of ~1 monolayer were formed by thermal oxidation of clean Si or SiGe surfaces under reduced pressure at lower temperature (<600 °C) [32–36]. As shown in Figure 3, the deposition of Si or Ge atoms on the ultrathin Si oxide film under ultrahigh vacuum (UHV) caused the following reactions: Si + SiO_2_→2SiO↑ or Ge + SiO_2_→GeO↑ + SiO↑, respectively. This resulted in the formation of nanowindows (NWs) less than 1 nm in size. As the deposition of Si or Ge continued, these atoms were trapped in the NWs, which caused spherical nanocrystals (or NDs) to form. When the NWs had formed sufficiently in the ultrathin Si oxide films, the NDs contacted the underlying Si(Ge) layer, though NWs resulting in the epitaxial growth of the NDs. If the substrate temperature was low during the formation of the NWs, the NW reaction did not occur sufficiently, which caused the NDs to grow non-epitaxially on the NWs.

**Figure 4.**
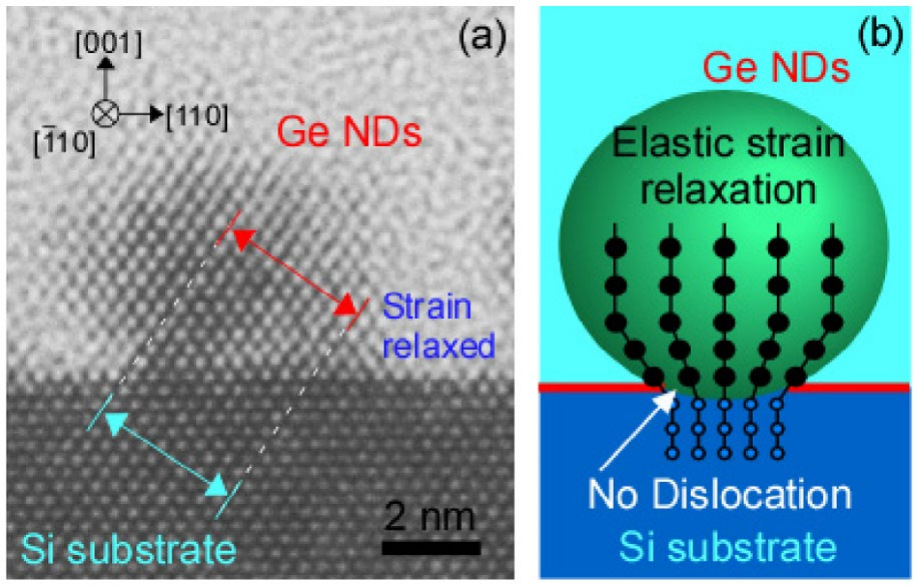
(a) Cross-sectional high-resolution transmission electron microscopy image and (b) the schematic of Ge NDs epitaxially grown on Si substrate. Reprinted (adapted) with permission from Nakamura et al. [49]. © 2011 American Chemical Society.

**Figure 5.**
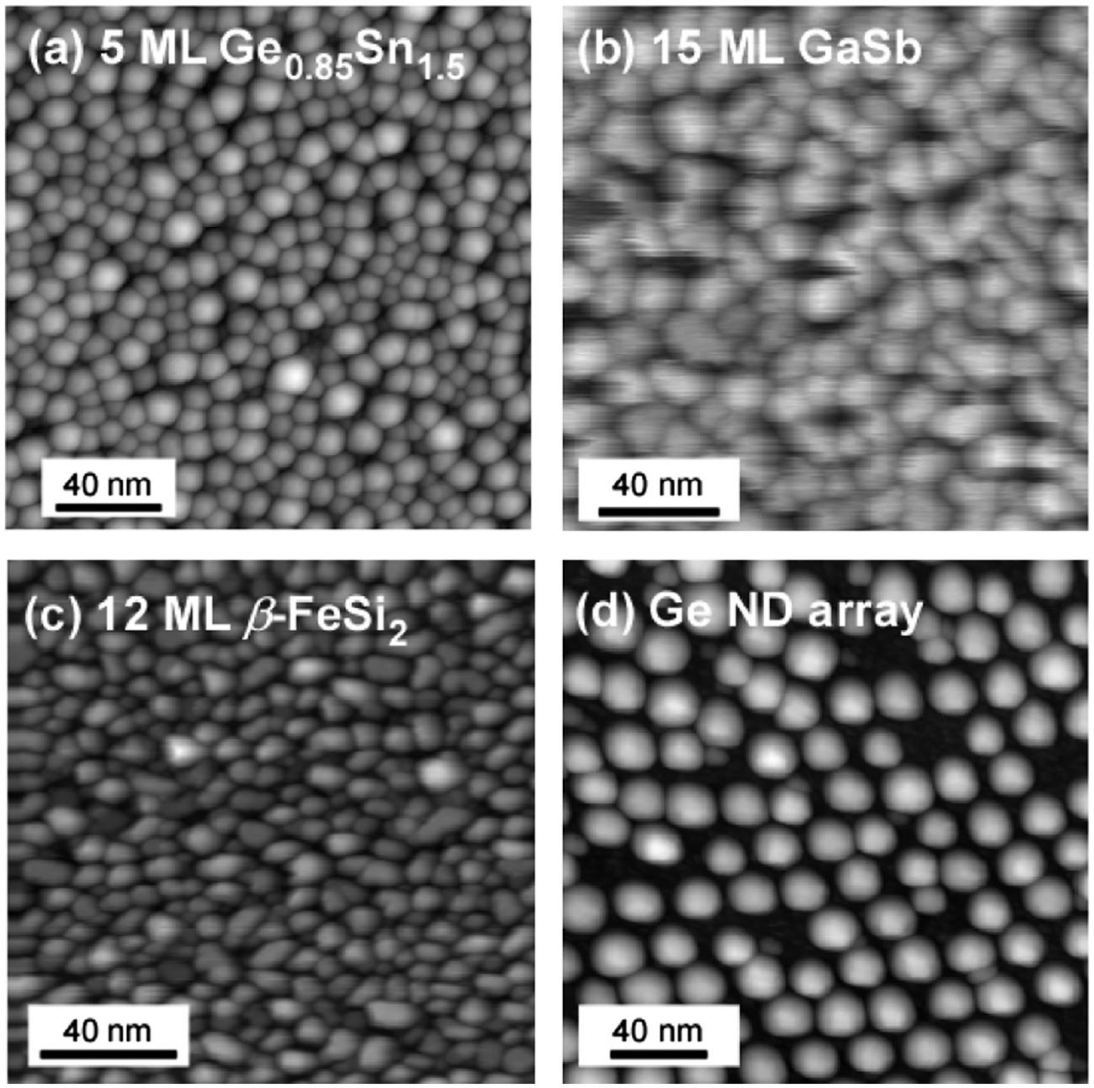
Scanning tunneling microscopy images of NDs formed by ultrathin Si oxide film technique. (a) 5-monolayer (ML) Ge_0.85_Sn_0.15_ NDs, (b) 15-ML GaSb NDs, (c) 12-ML *β*-FeSi_2_ NDs, and (d) Ge ND array.

The NDs exhibited high density, small size, and elastic strain relaxation when compared with conventional NDs. Conventional NDs of the group IV semiconductors are generally formed by Stranski–Krastanov (SK) growth mode [37–41]. SK NDs are formed by island growth after wetting layer growth. The island growth occurs for relaxation of the strain caused by lattice mismatch between the substrate and the growth material. These islands are called NDs or quantum dots. When Ge is grown on a Si substrate, the island density is low (~10^10^ cm^−2^) and the island size along the in-plane direction is large (~80 nm). Moreover, lattice mismatch dislocations occur during growth, and sometimes mixing of Si and Ge takes places. On the other hand, in the case of the ultrathin Si oxide film technique, because driving force of ND formation is not the strain relaxation, their characteristics were significantly different from those of the SK NDs. The ND density was ultrahigh (>10^12^ cm^−2^), which was determined by the density of the NWs which work as trapping sites of the deposited atoms resulting in spherical and ultrasmall (~ several nm) NDs growth on NWs. Additionally, the ultrasmall size of the NDs and the ultrasmall contact area between the NDs and the substrate though the NWs caused elastic strain relaxation without dislocations in the NDs (Figure 4). As a result, the NDs did not exhibit lattice mismatch dislocation and were highly crystalline. The ultrathin Si oxide film between the NDs and the underlying layers (mostly Si) minimized any mixing. This ND formation technique can be applied to a range of materials except Si or Ge (Figure 5) [42–46] by depositing different materials on the NWs following the initial deposition of Si or Ge on an ultrathin Si oxide film. Furthermore, when the positions of the NWs are controlled by self-organization techniques, the ND positions can also be controlled. Two-dimensional arrays of NDs can be formed by arranging the NWs on the ultrathin Si oxide film using a block copolymer (Figure 5(d)) [47].

**Figure 6.**
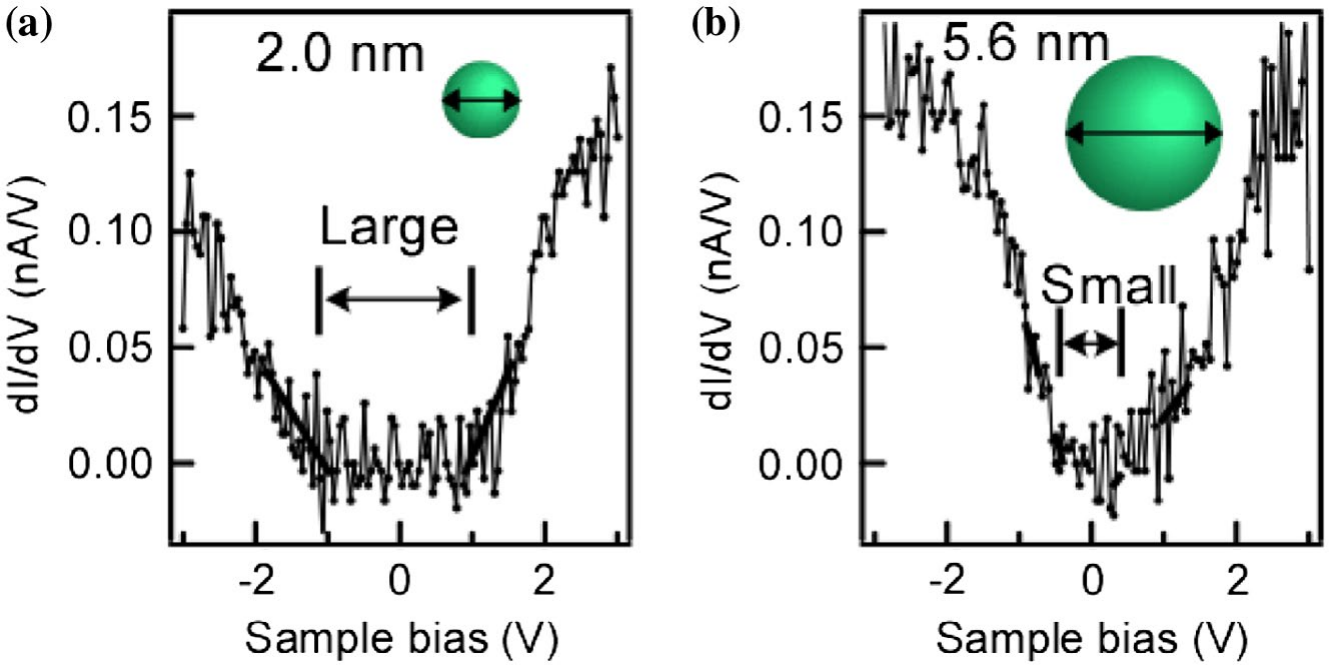
Scanning tunneling spectroscopy of Ge NDs with (a) small (2.0 nm) and (b) large (5.6 nm) diameters. Reprinted (adapted) with permission from Nakamura et al. [36]. © 2005 American Institute of Physics.

**Figure 7.**
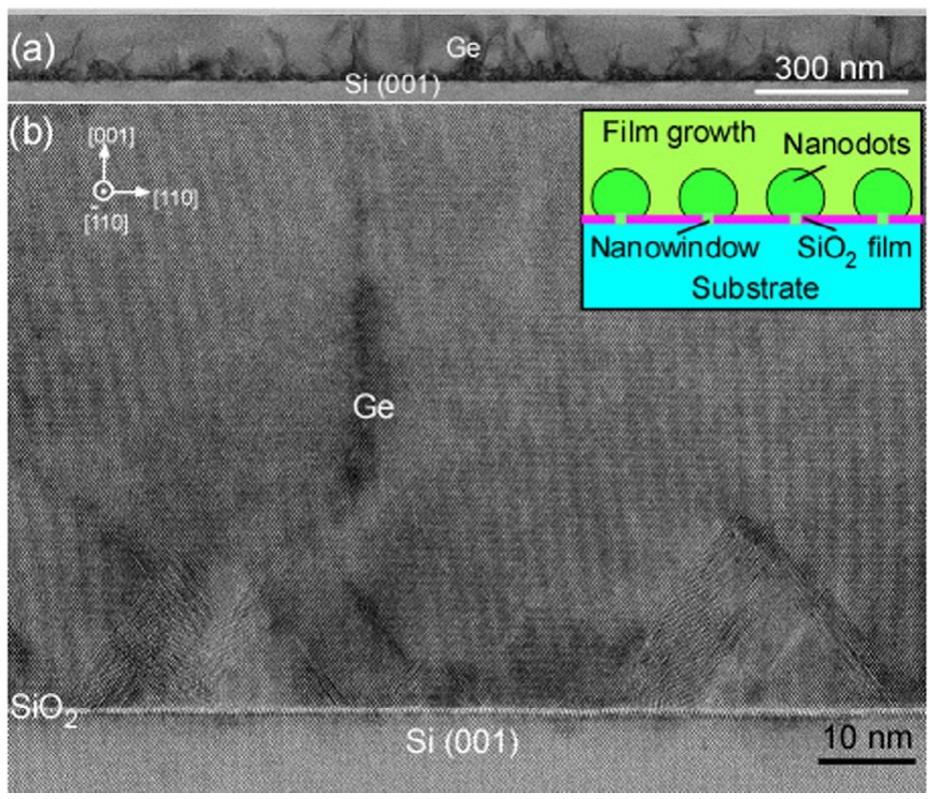
Cross-sectional HRTEM image of Ge films/Si formed by nanocontact epitaxy. (a) low and (b) high magnification. Reprinted (adapted) with permission from Nakamura et al. [49]. © 2011 American Chemical Society.

Since these NDs are of high quality, the change of density of states (DOS) caused by quantum confinement effects were observed successfully even at room temperature (Figure 6) [36,42,48]. The DOS change was observed directly using scanning tunneling spectroscopy of individual NDs, and not indirectly using photoluminescence of the ensembles. The energy bandgap of the NDs was dependent on their size (smaller size gave larger bandgap). These NDs are being studied for the development of a new heteroepitaxial growth method called nanocontact epitaxy [49,50]. High-quality NDs were used as seeds for heteroepitaxial growth. The lattice mismatch strain was relaxed within the ultrasmall NDs that contacted the substrate through the NWs, as shown in Figure 7. Importantly, various high-quality films were grown epitaxially using this technique. These results indicated that the inside and the interfaces of the NDs were of high quality.

## 3. The connected epitaxial Si nanodot structure

**Figure 8.**
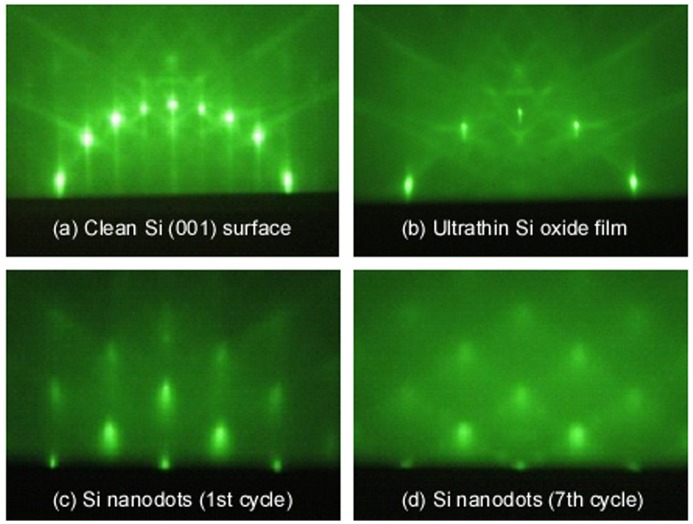
RHEED patterns of (a) clean Si surfaces, (b) ultrathin Si oxide film, and (c) and (d) Si nanodots ((c) 1st and (d) 7th cycles).

The structures of connected epitaxial Si NDs were formed by repeating the formation of Si NDs using the ultrathin Si oxide film technology. The fabrication process is described below. After Si (001) substrates were introduced into UHV of 2 × 10^−8^ Pa, clean Si surfaces were prepared by formation of a 100 nm Si buffer layer at 500 °C after degassing at 500 °C, as shown by a reflection high energy electron diffraction (RHEED) pattern (Figure 8(a)). Oxygen gas was introduced into the UHV chamber until the oxygen partial pressure reached 2 × 10^−4^ Pa to oxidize the clean surfaces for 10 min at 500 °C. This gave the ultrathin Si oxide film (Figure 8(b)). Subsequently, the epitaxial Si NDs were grown by depositing Si atoms on the ultrathin Si oxide film between 450 and 500 °C. These processes were repeated, which yielded the connected epitaxial Si ND structure.

**Figure 9.**
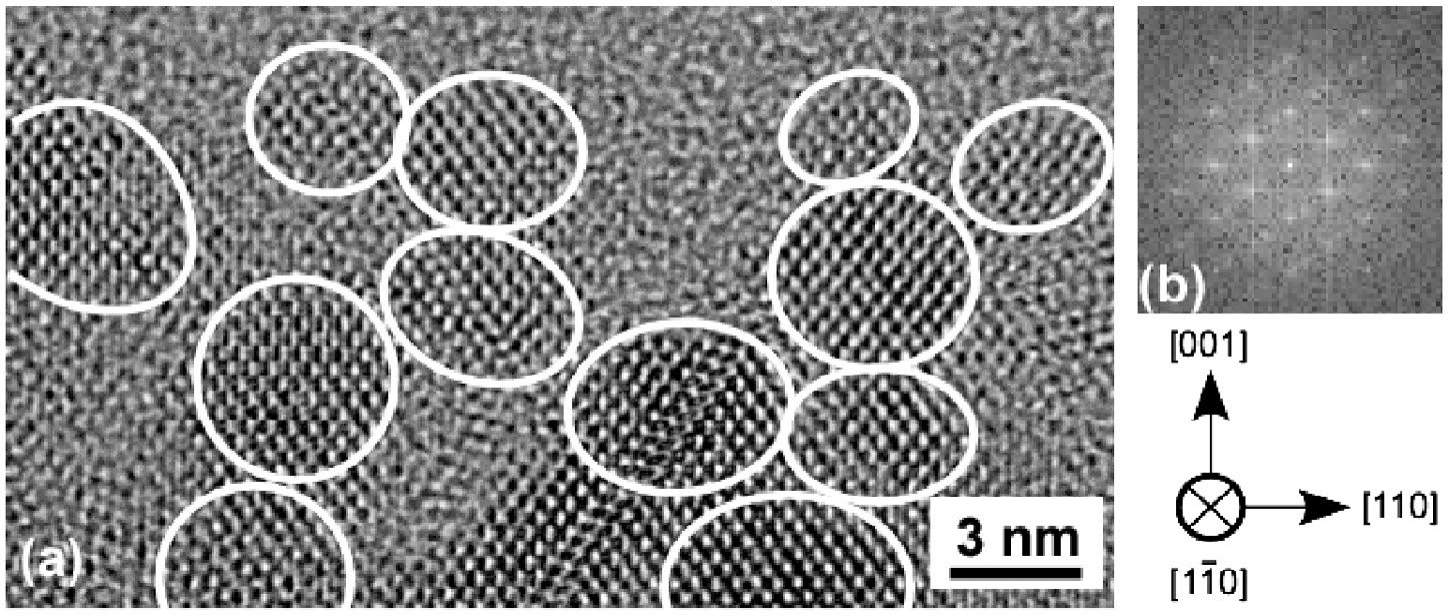
(a) HRTEM image of connected epitaxial Si NDs and (b) FFT pattern of Si NDs. Reprinted with permission from Nakamura et al. [30], (c) 2015 Elsevier.

The RHEED patterns of the Si surfaces, the ultrathin Si oxide film, and epitaxial Si NDs are shown in Figure 8. Clean Si(0 0 1)−(2 × 1) surfaces, amorphous surfaces of Si oxide film, and the epitaxial growth of Si NDs were confirmed. A cross-sectional, high-resolution transmission electron microscopy image (HRTEM) of the connected Si NDs that was several nm in diameter is shown in Figure 9(a). This revealed that NDs with a diameter of ~3 nm were connected to one another. Fast Fourier transform (FFT) patterns indicated that these Si NDs grew epitaxially (Figure 9(b)).

**Figure 10.**
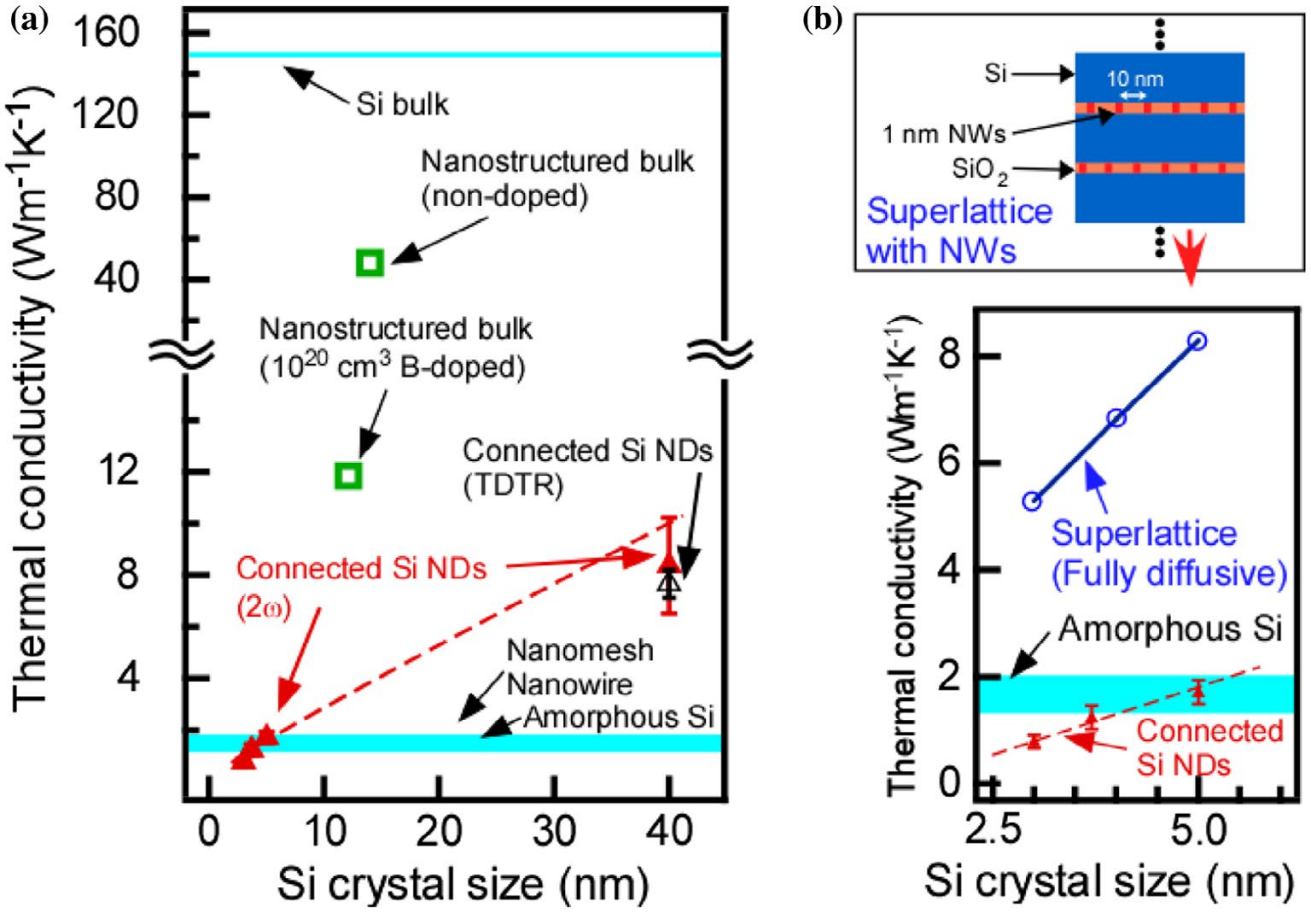
(a) Thermal conductivity of connected epitaxial Si NDs with the preceding Si nanostructure results, (b) enlarged graph of (a). Reprinted with permission from Nakamura et al. [30], © 2015 Elsevier.

The cross-plane thermal conductivity of these connected epitaxial Si NDs was measured using the 2ω method. In the 2ω method, Au films formed on the sample surfaces were Joule-heated by sinusoidal current and the modulated thermoreflectance signal at the Au films surfaces was detected using a lock-in amplifier to evaluate the thermal conductivity of the films. The detail of 2*ω* method has been written in the literature [30,51–55]. The thermal conductivity results are shown in Figure 10. The preceding results of Si materials are also displayed in Figure 10 for reference. These results revealed that the thermal conductivity declined as the size of the ND decreased. Remarkably, the NDs with a 3-nm diameter exhibited a thermal conductivity below the amorphous value, which is considered the minimum value in classical theory (amorphous limit). To determine whether this ultralow thermal conductivity could be explained using the conventional model, we computed the thermal conductivity of the Si/SiO_2_ superlattice, which has NWs similar to those of the Si NDs/ultrathin SiO_2_ films. In this calculation, the interfacial thermal resistance value was used in the conventional diffusive mismatch model. The calculated results are shown in Figure 10(b), which is an enlarged graph of Figure 10(a). This indicated that the ultralow thermal conductivity that was observed in the Si NDs was difficult to explain using the conventional model.

**Figure 11.**
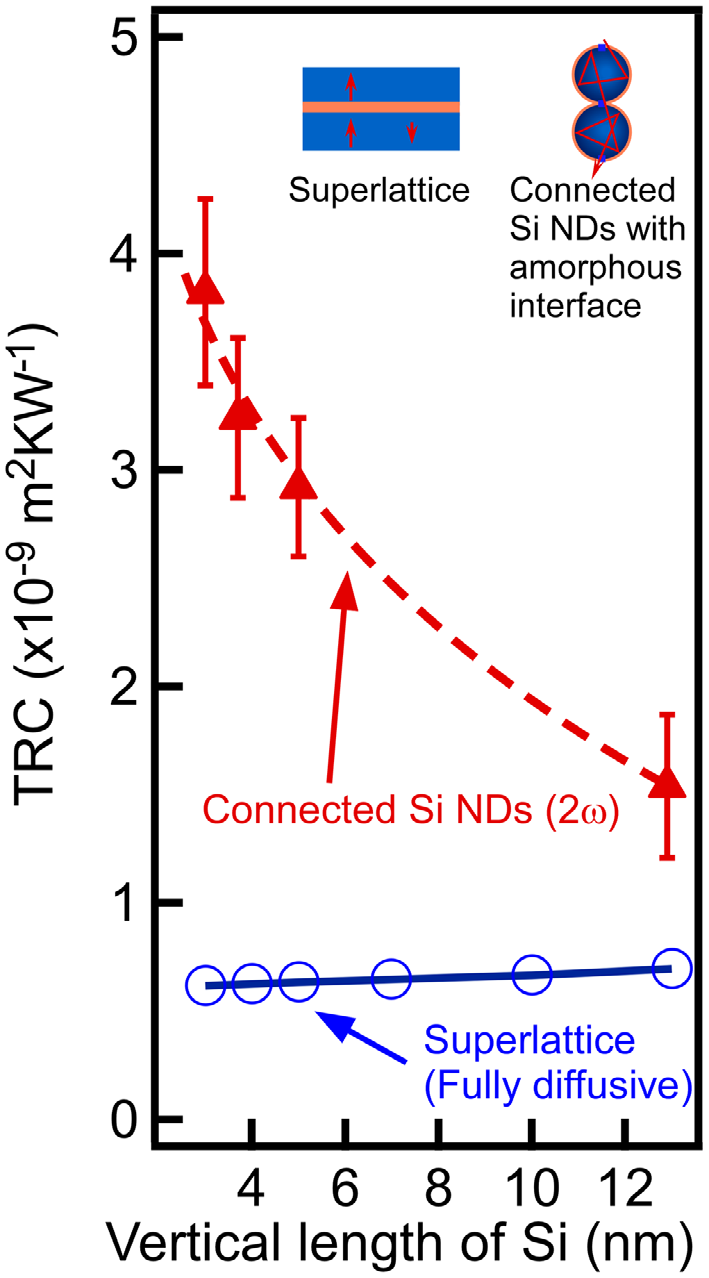
TRC of connected epitaxial Si NDs and the calculated TRC of Si/SiO_2_ superlattice. Reprinted with permission from Nakamura et al. [30], © 2015 Elsevier.

To investigate whether the low thermal conductivity was caused by the increased interface density accompanying a ND size reduction, we estimated the thermal resistance per cycle (TRC) by dividing the thermal resistance of the entire structure films by the number of individual ultrathin Si oxide films/Si NDs unit structures. The number was obtained by counting the unit structure (=ultrathin Si oxide film/Si ND) from the substrate to the film surface in the cross-sectional HRTEM images. The TRC results are shown in Figure 11. It revealed that TRC increased as the ND size decreased. Because the SiO_2_ was a monolayer thick and Si has a large thermal conductivity, the main contribution to the TRC was considered to be the interfacial thermal resistance in the Si oxide/Si NDs. In the conventional diffusive mismatch model, the interfacial thermal resistance is determined by the materials that form the interfaces and does not depend on the shape (radius of curvature) of the interfaces. However, in our connected ND structure, the TRC was dependent on the size of the Si NDs (i.e*.* the radius of curvature of the interface). This is a very remarkable fact. Therefore, the TRC of the Si/SiO_2_ superlattice that contained NWs (the schematic in Figure 10(b)) was calculated with the finite-element method using the diffusive mismatch model [56–58]. The conventional diffusive mismatch model cannot explain the large TRC of the connected ND structure (Figure 11). This demonstrated that phonon transport/scattering cannot be explained using conventional theory if the nanostructure is smaller than the mean free path of the phonons [5,59]. In this case, we have to consider the change of the phonon density of state in NDs, rather than phonon transport based on the mean free path of phonon. The phonon coherence effect similar to the phononic zone folding in the phononic crystal [60] might be observed in the present system with identical ND separation even though ND positions are random. Moreover, we have to consider the phonon frequency shift in NDs that can occur when the NDs are smaller than 10 nm. A small shift of 5–15 cm^−1^ was observed for 3–5 nm NDs in Raman experiments [61]. Then, in the present connected Si ND samples, a small variation of ND size causes the small energy difference of phonon among NDs. It can be difficult to observe the coherent effect at room temperature because there are the mismatch of phonon energy and the existence of the ultrathin SiO_2_ films among NDs in addition to the short coherent length of phonon. Then, the phonon energy mismatch and the ultrathin SiO_2_ films reduced the phonon transmittance probability, which can be a main mechanism for reduction of the thermal conductivity, rather than the coherence effect of phonon among NDs.

## 4. Si films containing the epitaxial Ge NDs

**Figure 12.**
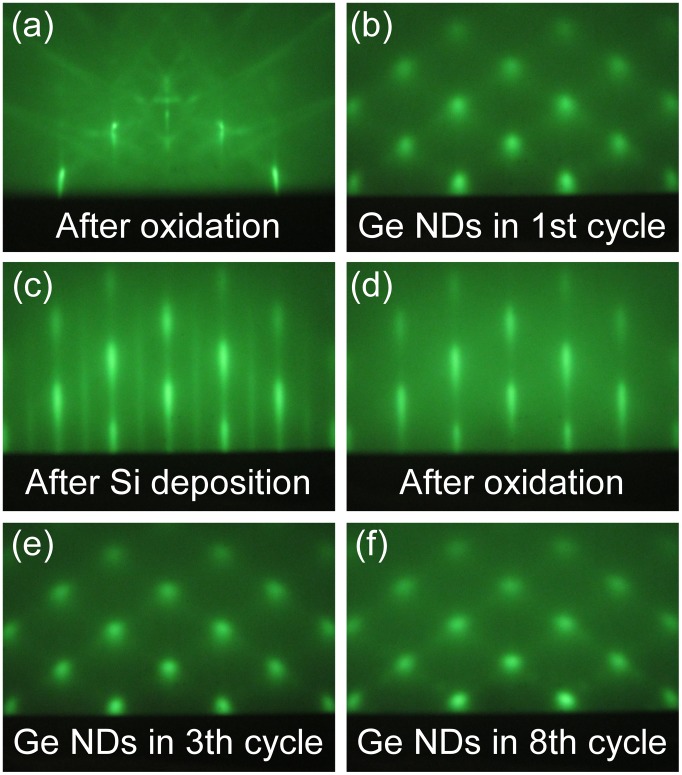
RHEED patterns of (a) ultrathin Si oxide film, (b)Ge NDs formed in 1st cycle process, (c) Si layers, (d) the ultrathin Si oxide film on Si layer, (e) and (f) Ge NDs formed in (e) 3th and (f) 8th cycles.

**Figure 13.**
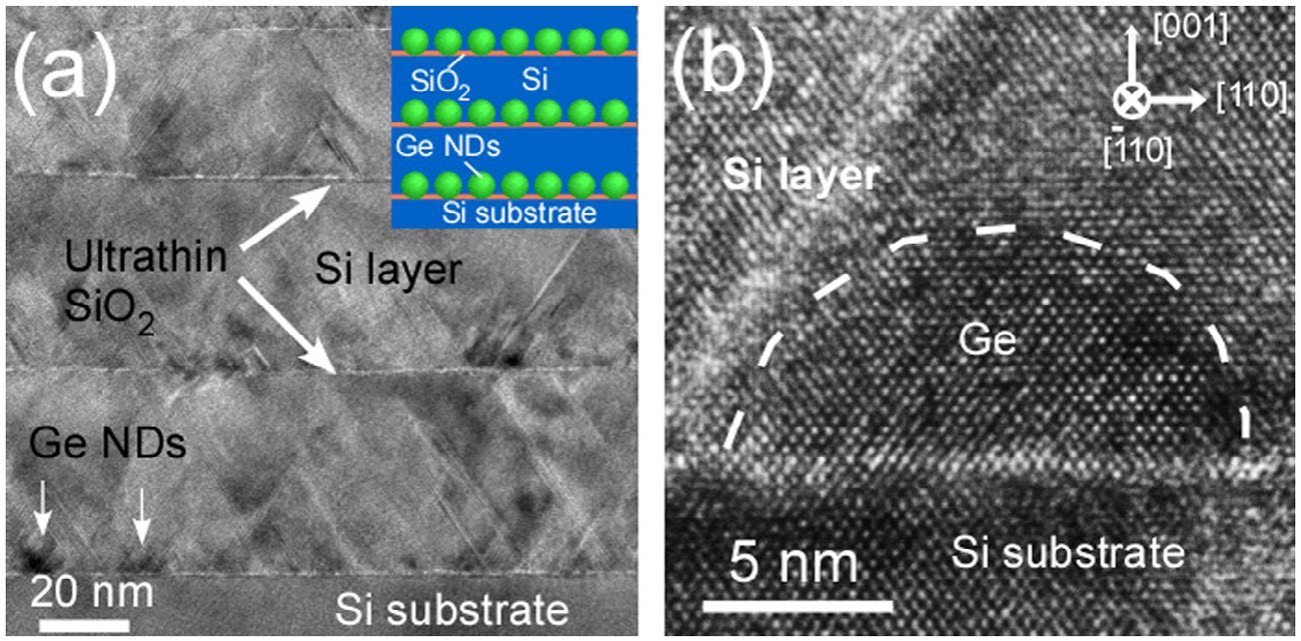
Cross-sectional HRTEM image of Si films containing Ge NDs (stacking structure of Ge NDs/Si) formed by ultrathin SiO_2_ film technique (a) and high magnification image near the interface (b). The inset in (a) is a schematic of Si films containing Ge NDs. Reprinted (adapted) with permission from Yamasaka et al. [7]. © 2015 Springer Nature.

The Si films containing epitaxial Ge NDs were formed by repeating epitaxial growth of the Si layers for the electrical conduction layer and Ge NDs for the phonon scattering bodies using the ultrathin Si oxide film technology. In this process, first, the ultrathin Si oxide films were formed by oxidation of clean Si surfaces as described in Section 3. Epitaxial Ge NDs were then formed by depositing Ge on the ultrathin Si oxide films between 450 and 500 °C. Subsequently, epitaxial Si layers were then formed on the Ge NDs by deposition of Si at 400 °C. The repetition of the formation of the ultrathin Si oxide films, Ge NDs, and Si layers yielded the desired structure (Figure 2(b)). The RHEED patterns of this structure are shown in Figure 12. It was confirmed that the Ge NDs and the Si layers were grown epitaxially. When the formation process was repeated, the epitaxial growth continued, although the crystallinity decreased. The cross-sectional HRTEM image and a schematic of this nanostructured film are shown in Figure 13(a). A high magnification HRTEM image near the interface of the ultrathin Si oxide film is shown in Figure 13(b). This indicated that the Ge NDs were formed on the Si layers. These results confirmed that the Si films containing the Ge NDs were formed as designed.

**Figure 14.**
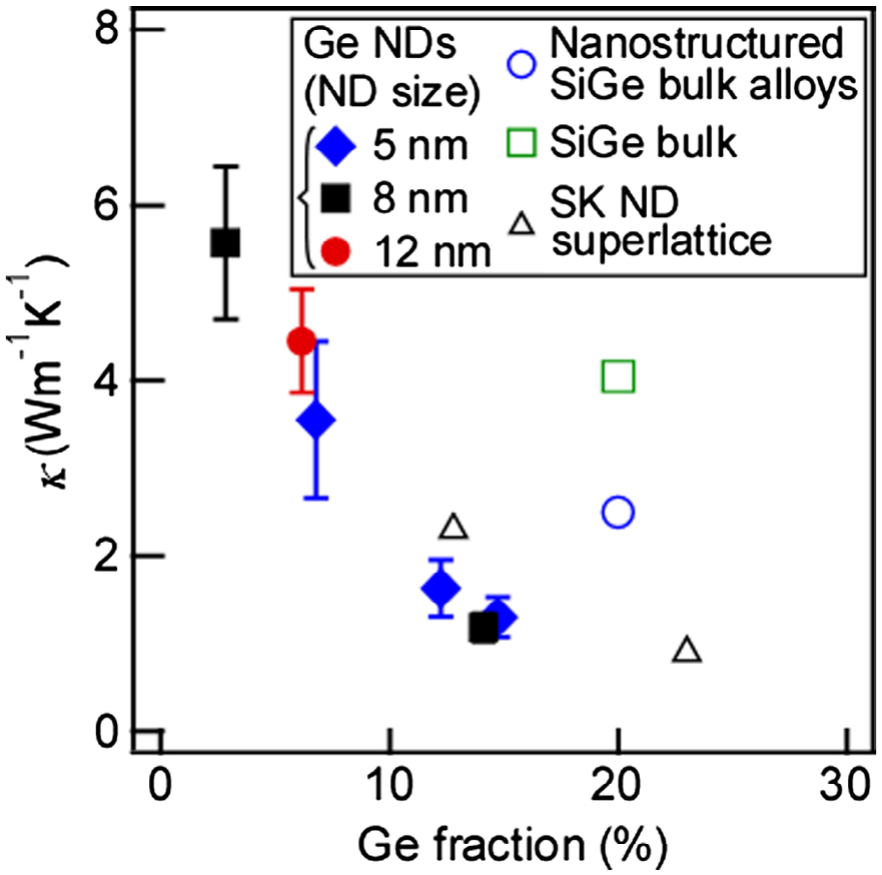
Thermal conductivity of Si films containing Ge NDs (stacking structure of Ge NDs/Si) with the preceding results. Reprinted (adapted) with permission from Yamasaka et al. [7]. © 2015 Springer Nature.

The cross-plane thermal conductivity of the Si films containing the epitaxially grown Ge NDs was measured using the 2ω method. The detail of this measurement method is written in the previous section. The results are shown in Figure 14 (solid marks) [7]. For reference, the results of SiGe bulk material [11,62], SiGe nanostructured bulk material (sintered SiGe including nanocrystals) [62], and SK NDs superlattice [40,41], are also shown as open marks. This comparison showed that the Si films containing the epitaxially grown Ge NDs had a significantly lower thermal conductivity than those of the reference materials.

**Figure 15.**
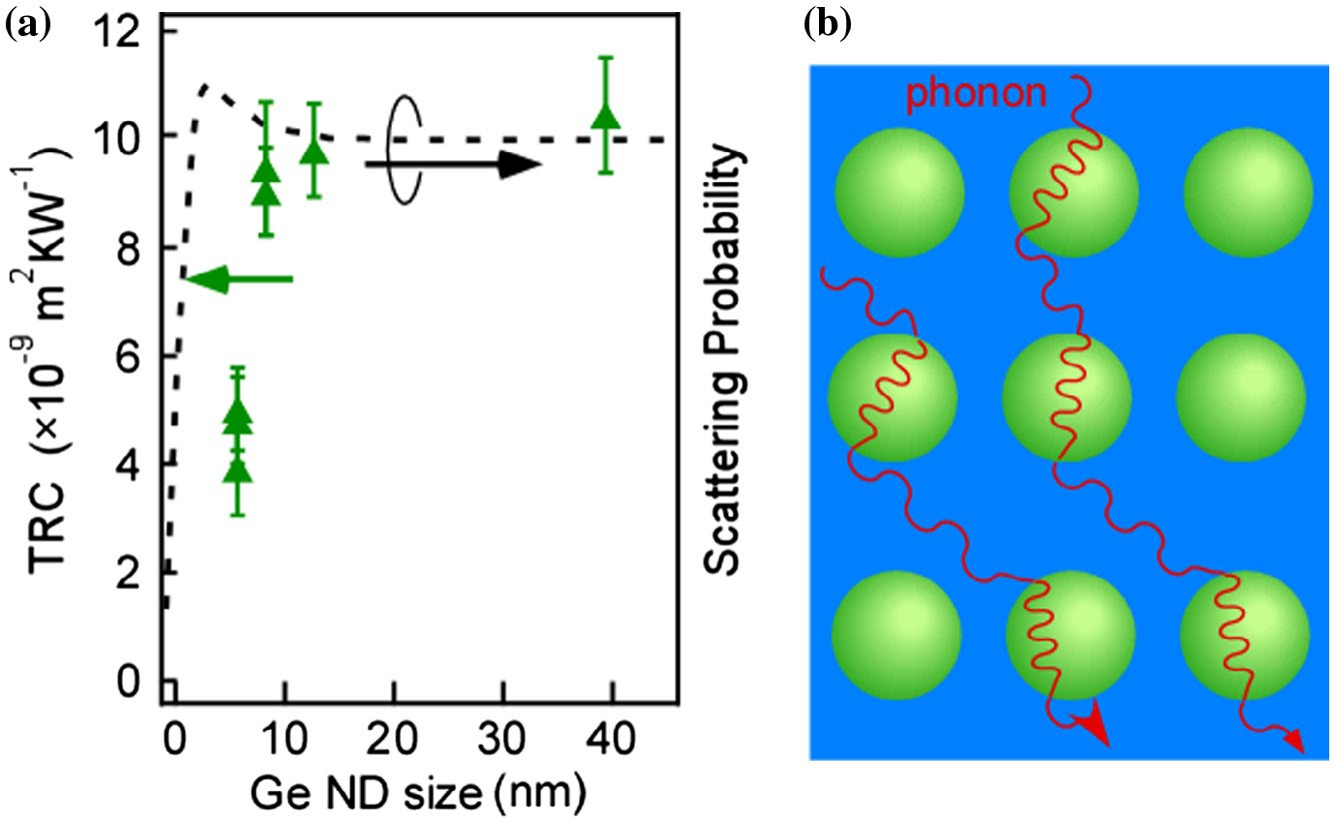
The Ge ND size dependence of the TRC in Si films containing Ge NDs with the rough tendency of phonon scattering probability (the dashed line) (a) and the schematics of phonon scattering and transports (b). Reprinted (adapted) with permission from Yamasaka et al. [7]. © 2015 Springer Nature.

This thermal conductivity reduction was observed even when the Si layers are rough, namely non-periodic ND structures in vertical direction [7]. Therefore, we considered that this reduction does not come from the phononic folding effect. To investigate the effect on the reduction of thermal conductivity in this nanostructure, we estimated the TRC by dividing the thermal resistance of the entire structure films by the number of Si/Ge NDs/SiO_2_/Si unit structures. As shown in Figure 15, this structure exhibits high TRC which was dependent on size of the NDs. This high TRC value cannot be explained by the interfacial thermal resistance in the diffusion mismatch model, the thermal resistance of Si and Ge, and the thermal resistance of the SiO_2_ with a thickness of one monolayer. The dependence of the TRC on the size of the NDs could be simply explained by the interfacial area between the Ge NDs and Si being dependent on the size of the Ge ND. However, when the estimating surface area of the Ge NDs (which is equivalent to the interfacial area between ND and Si), it was found that the interfacial area did not show a strong dependence on ND size within the ND size range in Figure 15. This indicated that the high thermal resistance and dependence on the size of the Ge NDs could not be explained by the conventional increase effect of interfacial area. We propose that the observed TRC behavior may occur because of a wave motion in the scattering of the phonons on the Ge NDs. When the scattering probability of a wave motion in a material that includes scattering particles is calculated, the probability is dependent on the size of scattering particles. When the characteristic phonon wavelength in Si is defined as ~1 nm [5,59], the scattering probability roughly had the tendency shown by the dashed line in Figure 15(a) [63]. Because this approach is analogous to the optical scattering that corresponds to Mie and Rayleigh scattering, the similarity between TRC and scattering probability implies that the observed scattering was caused by a wave motion of the phonons (Figure 15(b)). Now, it is needed to validate this proposed model in the future. In this scattering framework, the scattering probability becomes lower when ND size is smaller than phonon wavelength (~1 nm in this case). This means that ND can scatter only phonon with longer wavelength than ND size effectively. On the other hand, in the case of larger NDs than coherent length of phonon, such scattering does not occur. Let’s consider the mean free path of phonon related to wavelength. According to the literatures about the accumulative thermal conductivity of Si [64–66], where the accumulative thermal conductivity was reported as a function of mean free path. In the present Si films containing Ge NDs, phonon with relatively longer mean free path is effectively scattered. Then, according to the graph of the accumulative thermal conductivity vs mean free path [64–66], further reduction of thermal conductivity can be expected when the scattering centers with various scales are introduced, which can scatter many phonons with various mean free paths.

**Figure 16.**
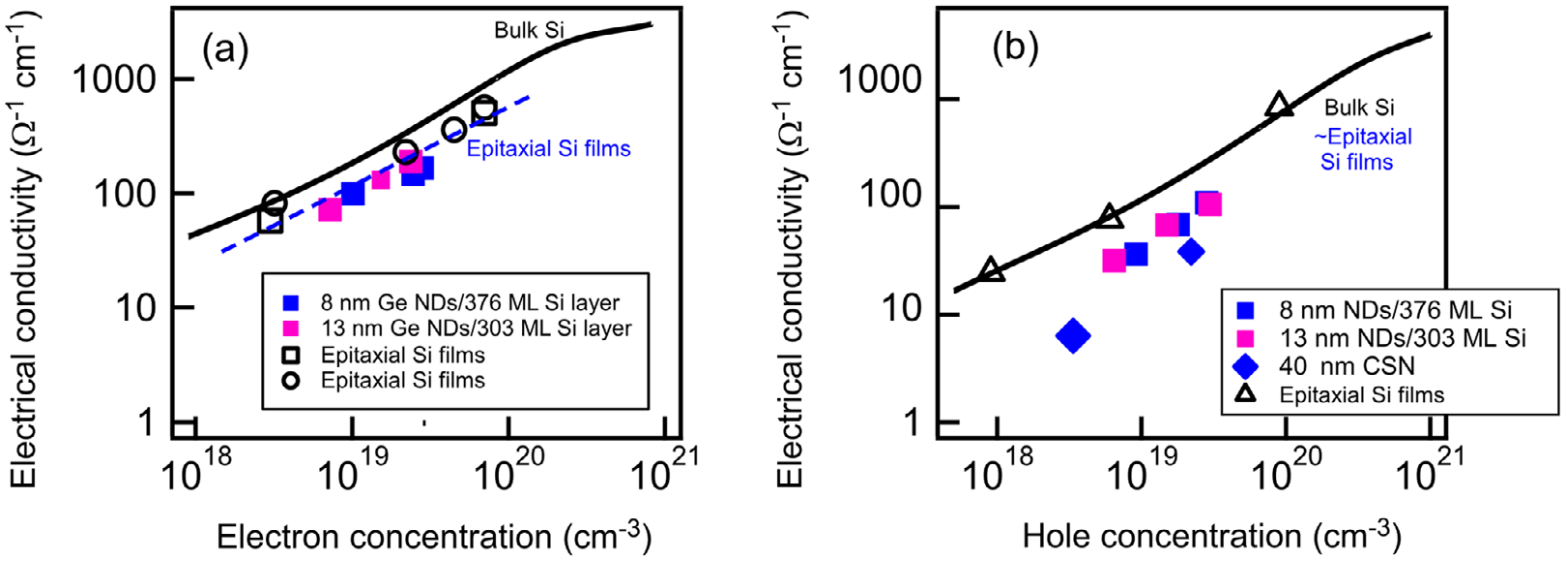
The electrical properties of Si films containing Ge NDs. P-doped (*n*-type) (a) and B-doped (*p*-type) samples (b). Results of epitaxial Si films in literatures are also shown for references (open square, circle, triangle marks are from Refs. [68–70], respectively). Calculated lines for bulk Si are also taken from Ref. [67]. Dotted line in (a) is an eye-guide.

To investigate the electrical properties of these nanostructured thin films, ion implantation was performed using P, and then the electrical properties were evaluated by Hall effect measurements [31] as shown in Figure 16. The dependence of the electrical conductivity on electron concentration revealed that the conductivity of n-type samples was slightly lower than that of bulk Si [67], but was almost the same as Si thin films grown on Si substrates [68,69] while the electrical conductivity of p-type samples on hole concentration is smaller than those of the bulk Si [67] and epitaxial Si films [70].This indicated that the phonons scattered on the ND interfaces in the Si films containing epitaxially grown Ge NDs, but the effect of electron scattering on the NDs was small. It was considered that the small band offset between Si and Ge at the bottom of the conduction band would decrease interface scattering probability of electron unlike hole conduction with large valence band offset. However, we need to investigate the energy barrier at the interface because Si layers on Ge NDs can be strained near these interfaces. Alternatively, the scattering at the interfaces may not affect the electron mobility significantly because the major scattering processes in the regions of high carrier concentration are caused by ionized impurities. In any case, the reduction of the electrical conductivity at the ND interfaces was small indicating almost no degradation of electric properties even when the NDs exist.

We should discuss about the anisotropic effect of the structures on thermoelectric properties because *κ* and *σ* were measured at the cross- and in-plane directions, respectively. In the general superlattice structures where flat layers are stacked alternately, the electrical anisotropy in *σ* was reported [71]. On the other hand, the isotropic thermoelectric properties were reported in the nanostructured bulk alloys with randomly positioned grains [72]. In the present Si films containing Ge NDs, Ge NDs were placed on the flat Si layers periodically arranged along the cross-plane direction in the case of large Si layer thickness (>300 monolayers) [31], which indicating the anisotropic structure. On the other hand, Ge NDs were randomly positioned due to the Si layer roughness in the case of small Si layer thickness (<~70 monolayers); namely the structure becomes quasi-isotropic as reported in the previous paper [31]. Electron mobility in both cases of thin and thick Si layers are almost the same as those of the epitaxial Si films without Ge NDs, indicating that electrical conduction anisotropy caused by Ge ND existence is very weak [31]. The lack of electrical conduction anisotropy can be explained by the weak electron scattering at the Si/Ge NDs which is discussed above. Therefore, we expected high *σ* and low *κ* can be expected at the same direction basing on such nanostructure strategy, but precise *ZT* estimation cannot be done unless the direct measurement of the two values at the same directions is carried out.

## 5. Summary

We have proposed the guideline for the independent control of heat flow and electric current by introducing well-controlled, nanostructured interfaces. Using an ultrathin Si oxide film technique, structures that consisted of connected epitaxial Si NDs or Si films containing epitaxial Ge NDs were formed. The thermal and electrical conductivity of these structures were measured. The thermal conductivity of both structures including the NDs was reduced dramatically. The structures of connected epitaxial Si NDs (~3 nm in diameter) exhibited a thermal conductivity that was below the amorphous limit. We proposed that the reduction of the thermal conductivity observed in the Si films containing epitaxial Ge NDs was caused by efficient phonon wave scattering at the Ge NDs. Conversely, electron scattering at the interface between the Ge NDs and Si did not contribute significantly to the reduction in the electron mobility. These results demonstrated that the drastic reduction of thermal conductivity and the preservation of high electrical conductivity can be done simultaneously using well-controlled, nanostructured interfaces based on the theory of nanostructure physics, which has the potential to contribute significantly to the successful fabrication of Si-based thermoelectric materials.

## Disclosure statement

No potential conflict of interest was reported by the author.

## Funding

This study was supported by PRESTO, Japan Science and Technology Agency (JST); Grant-in-Aid for Scientific Research A [grant number 16H02078]; a Grant-in-Aid for Exploratory Research [grant number 15K13276], JST CREST Program.
